# FLEXGRID – A novel smart grid architecture that facilitates high-RES penetration through innovative flexibility markets towards efficient stakeholder interaction

**DOI:** 10.12688/openreseurope.14109.1

**Published:** 2021-10-22

**Authors:** Nikolaos Efthymiopoulos, Prodromos Makris, Georgios Tsaousoglou, Konstantinos Steriotis, Dimitrios J. Vergados, Alireza Khaksari, Lars Herre, Victor Lacort, German Martinez, Elena Leal Lorente, Robert Gehrcke, Matin Bagherpour, Gesa Milzer, Bryan Pellerin, Farhan Farrukh, Malte Thoma, Tonci Tadin, Maria-Iro Baka, Christina Papadimitriou, Andreas Kyprianou, George E. Georghiou, Hrvoje Pandzic, Domagoj Badanjak, Spyros Chatzivasileiadis, Eléa Prat, Mihai Calin, Tara Esterl, Filip Pröstl Andrén, Emmanouel Varvarigos

**Affiliations:** 1 Institute of Communication and Computer Systems, National Technical University of Athens, Athens, 15773, Greece; 2 University of Western Macedonia, Kastoria, Greece; 3 Technical University of Denmark, Kgs Lyngby, Denmark; 4 Etra Investigacion Y Desarollo Sa, Valencia, Spain; 5 Nord Pool Consulting, Oslo, Norway; 6 Nodes As, Kristiansand, Norway; 7 Smart Innovation Norway, Halden, Norway; 8 Bnnetze Gmbh, Freiburg Im Breisgau, Germany; 9 Hops, Zagreb, Croatia; 10 University of Cyprus, Nicosia, Cyprus; 11 University of Zagreb, Zagreb, Croatia; 12 Austrian Institute of Technology, Wien, Australia

**Keywords:** demand response, flexibility markets, optimization, smart grids, flexibility services, digital energy services, network-aware market clearing, market-aware grid management, smart grid management

## Abstract

The FLEXGRID project develops a digital platform designed to offer Digital Energy Services (DESs) that facilitate energy sector stakeholders (i.e. Distribution System Operators - DSOs, Transmission System Operators - TSOs, market operators, Renewable Energy Sources - RES producers, retailers, flexibility aggregators) towards: i) automating and optimizing the planning and operation/management of their systems/assets, and ii) interacting in a dynamic and efficient way with their environment (electricity system) and the rest of the stakeholders. In this way, FLEXGRID envisages secure, sustainable, competitive, and affordable smart grids. A key objective is the incentivization of large-scale bottom-up investments in Distributed Energy Resources (DERs) through innovative smart grid management. Towards this goal, FLEXGRID develops innovative data models and energy market architectures (with high liquidity and efficiency) that effectively manage smart grids through an advanced TSO-DSO interaction as well as interactions between Transmission Network and Distribution Network level energy markets. Consequently, and through intelligence that exploits the innovation of the proposed market architecture, FLEXGRID develops investment tools able to examine in depth the emerging energy ecosystem and allow in this way: i) the financial sustainability of DER investors, and ii) the market liquidity/efficiency through advanced exploitation of DERs and intelligent network upgrades.

## Plain language summary

Future smart grids require effective interaction between energy markets and grid management systems in order to introduce new services that support a high share of renewable energy. The European Union’s vision outlined in new directives highlights the need for:

1. A high share of distributed Renewable Energy Sources (RES) that help a transition to a carbon-emission free society
2. The liberalization of energy markets which provides opportunity for new stakeholders, new business models and innovative energy services
3. Economically sustainable investment in flexible energy assets

The Horizon 2020 research project FLEXGRID aims to answer to the following critical questions:

- How can distribution system operators deal with the new operational challenges caused by a large share of renewable generation?
- How can system operators avoid costly grid reinforcement by efficiently utilizing flexibility?
- How can we develop economically sustainable business models for the emerging smart grid ecosystem?
- How can small energy prosumers participate in emerging flexibility markets?
- How can we best align the various roles and unique interests of the energy market stakeholders?

These are only some of the many questions that are examined and tested beyond theoretical discussion. FLEXGRID’s vision is to bridge the gap between reliable grid operation and flexibility market efficiency by developing advanced mathematical models and algorithms that enable a two-way cooperation: Market-aware distribution grid operation and Network-aware market operation. The idea is simple: to build an online flexibility marketplace centered smart grid architecture where flexibility buyers can easily buy services from flexibility suppliers!

## I. Introduction

The large-scale integration of Distributed Energy Resources (DERs) such as PV/Wind generation (RES), Electric Vehicles (EVs), Energy Storage Systems (ESS) and Demand Side Management (DSM) equipment in distribution networks poses new challenges and opportunities for the power sector, as stated in the EU Clean Energy Package. In this context, the FLEXGRID project investigates several constraints related to the current smart grids architecture that prevent large scale DER integration in distribution networks.

The first reason is that Distribution System Operators (DSOs) use conservative constraints in distributed DER installation to ensure reliable and secure operation of their network. The root cause of this conservatism is the inability of DSOs to, dynamically and accurately, monitor and manage their networks. The development of a dynamic and accurate Distribution Network (DN) monitoring system and of an efficient and dynamic DN management system would therefore be the first step towards mitigating this conservatism.

A second reason is that even in cases where DSOs dispose Distribution Management Systems with appropriate DN monitoring capabilities, the lack of intelligence that would allow the efficient and dynamic interaction with DER operators (i.e. RES producers, retailers, flexibility aggregators) hinders DER investments. Consequently, the DN-related costs remain high (usually driven by DN upgrades with high Capital expenditures (CAPEX). In this context, FLEXGRID develops advanced DESs relevant to the operation of Distribution Level Flexibility Markets (DLFMs), aiming to facilitate the efficient management of DNs and the reduction of distribution network management cost.

Another issue tackled in FLEXGRID is the inefficient investment planning and management of DER assets. To this end, FLEXGRID evolves existing smart grid architectures, which are not able to provide information related to the electricity grid topology and the market conditions to DER investors, by addressing the aforementioned shortcomings. Under this perspective, FLEXGRID exploits: i) topology and monitoring information from the networks that it manages and ii) data analytics from the market that it operates in order to provide DESs useful for the design of optimal DER investment strategies and optimal DER portfolio management.

Beyond these innovation levels, FLEXGRID copes with a major inefficiency in today’s smart grids, which is the lack of interaction between Transmission System Operators (TSOs) and DSOs. Today, the TSO is the main actor procuring flexibility from flexible units to ensure system stability. However, in the future, DSOs are expected to procure flexibility to solve issues in their networks, too. As DSOs and TSOs might use the same sources of flexibility, this flexibility should ideally be used in a coordinated way. Different market-based and non-market-based approaches can be used by the TSO and the DSOs for the coordination of flexibility ^1^. When using flexibility to cope with a given grid operation challenge, this might have an impact on other grid operation aspects. For example, the activation of DN-level flexibility for system balancing by the TSO might cause congestion in the distribution grid. Another example is the activation of DN-level flexibility by a DSO to solve a local congestion problem, which may cause higher re-dispatch costs at the transmission network (TN) level.

Another problem of ineffective TSO/DSO interaction in today’s smart grids is exhibited by the suboptimal economic dispatch decisions. These are often made by the DSOs or their issuing dispatch orders to DER operators that: i) are infeasible, due to DN constraints, and/or ii) in conflict with dispatch instructions sent by the DSO to end energy prosumers. DSOs, on the other hand, have observability into distribution system operations but, to date, have little to no experience in creating economically optimal system operations. Moreover, DSOs have little or no observability into transmission system conditions, and, in many cases, into the investment or operation decisions of DER owners. As a result, DSOs (and, equally often, TSOs) lack knowledge about the DER and DSM potential, which may act in support of system operations. This has led to a variety of discussions over how to coordinate DSO operations with consumers, DER operators, and TSOs as analyzed in 2 and in USEF Workstream.

In this new landscape, and in order to alleviate these architectural inefficiencies and immaturities of existing smart grids, FLEXGRID focuses on four major research gaps.

The first research gap examines the operation of existing energy markets and the evolution of their architectures in depth by focusing on the interaction between TSOs and DSOs. It also unfolds around the development of advanced market clearing and pricing algorithms able to adequately model the underlying distribution system and ensure market efficiency by considering modern DER models (e.g., ESS, DSM, etc.).

The second research gap spans around the development of DESs towards efficient aggregation of end user’s DERs. This alternative facilitates their optimal and parallel usage of their capacity in multiple energy markets according to FLEXGRID’s innovative energy market architecture.

The third research gap relates to the development of DESs that contribute to the optimal operation of DERs and the advanced planning of DER investments according to a sophisticated and data driven examination of: i) FLEXGRID’s innovative markets, ii) DN/TN topology, and iii) competition.

The fourth research thread focuses on the monitoring and management of the transmission and the distribution networks in smart grids and in resolving of problems related with the grid upgrades with respect to market power mitigation and flexibility exploitation. It co-optimizes networks upgrades and flexibility investments.

In the rest of this position paper, we present at a high level the innovative architecture of FLEXGRID and the major DESs that it develops. Section II depicts the high-level FLEXGRID architecture together with three innovative energy market architectures, which offer advanced interaction between TN- and DN-level markets. Section III presents the novel FLEXGRID DESs offered to various market stakeholders of FLEXGRID ecosystem. Finally, section IV concludes this position paper by outlining the major FLEXGRID goals and ongoing work.

## II. Methods

### 1. Implementation of flexgrid architecture

**Figure 1.**
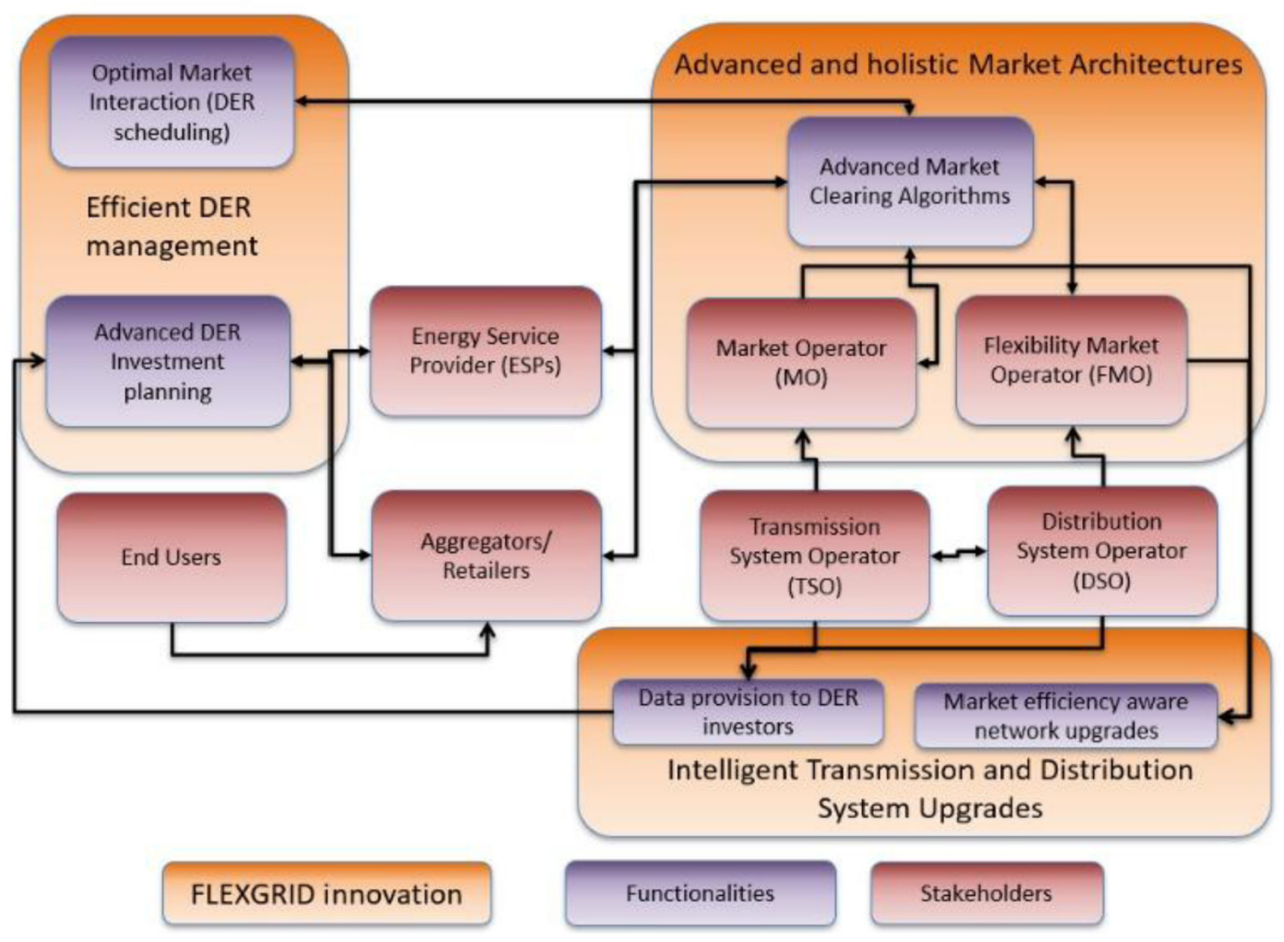
High-level functional architecture of FLEXGRID.

Briefly, FLEXGRID focuses on all the energy sector stakeholders in modern smart grids and proposes an innovative smart grid management architecture through which they can interact efficiently in order to optimally plan and operate their grids (transmission and distribution networks) and their DERs. Figure 1 depicts the high-level functional architecture of FLEXGRID.

From the grid management perspective, FLEXGRID develops innovative energy market architectures able to ensure the optimal use of DERs through advanced market clearing algorithms (dispatch and pricing). In order to achieve this, the proposed architecture guarantees an efficient interaction between TSOs and DSOs. More specifically, the Market Operator (MO, which is responsible for the efficient and network-aware market clearing at TN-level) interacts with Flexibility Market Operator(s) (FMOs, which are responsible for the efficient and network-aware market clearing at DN-level).

**Figure 2.**
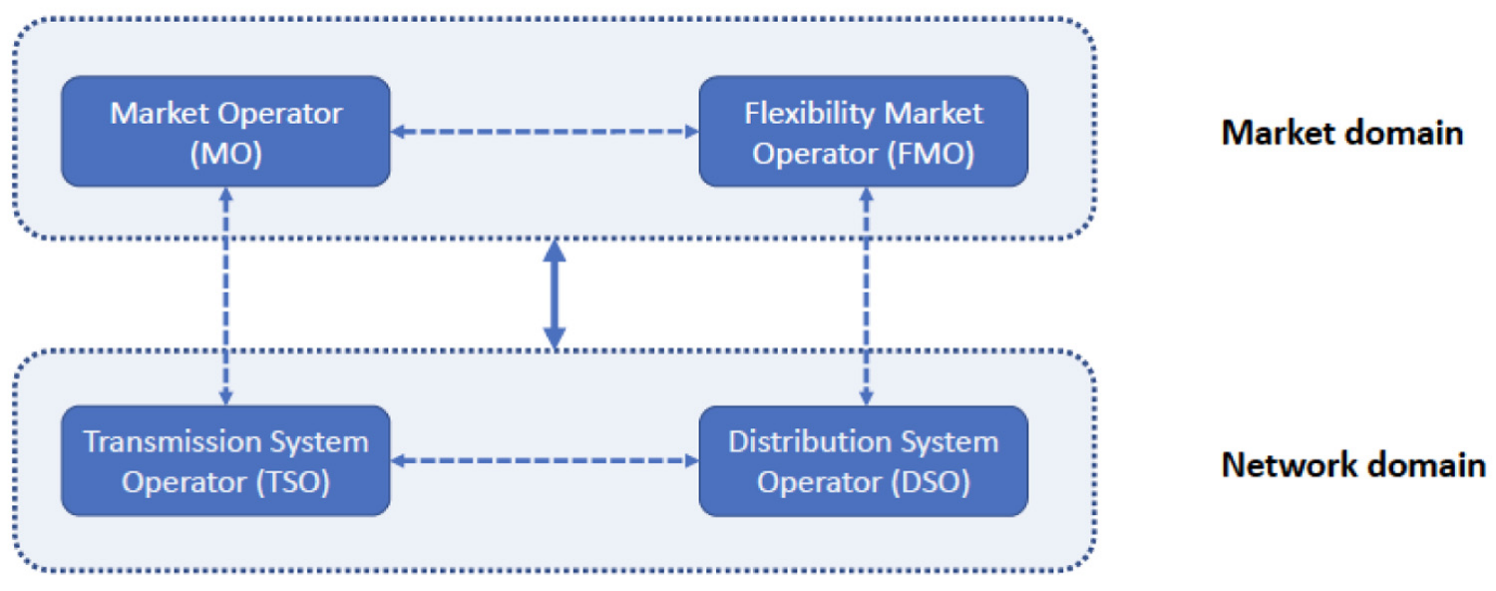
MO-FMO collaboration for better overall market efficiency outcomes and TSO-DSO collaboration for better operation outcomes for the electricity grid as a whole.

To develop innovative energy market architectures, FLEXGRID considers the “market” domain, in which the MO and FMO collaborate to improve operation and coordination of the wholesale (TN-level) and local (DN-level) energy markets, as shown in the Figure 2. Meanwhile, in the “network” domain, the TSO-DSO collaboration achieves improved network operation outcomes. The goal of FLEXGRID is to provide attractive trade-offs between optimal market and network operations, i.e., between economic efficiency and reliability, in a future with highly distributed RES penetration scenarios.

From the perspective of a DER investor, FLEXGRID accurately models modern energy sector stakeholders, often referred to as Energy Service Providers (ESPs), which are competitive stakeholders owning various types of DER assets that interact with the DN and/or TN. ESPs are able to participate in various energy markets to ensure their financial sustainability by providing competitive energy services (such as trade of capacity, energy and flexibility services). Thus, FLEXGRID provides to ESPs the necessary intelligence for optimal and robust planning of their investments in DERs and to schedule their operation in an efficient way according to the various market needs. ESPs own DERs which are compatible (in timing and bidding format) with today’s existing markets, so these DERs can be introduced easily in these markets.

From the perspective of the interaction between modern smart grids and end users, FLEXGRID focuses on the effective DN level aggregation of flexibility assets from end users and their efficient exploitation. FLEXGRID develops management tools for a specific type of ESP (denoted as FSP/Aggregator). FLEXGRID provides intelligent retail pricing and auction algorithms to independent FSPs to efficiently interact with end users through a B2C flexibility market. In addition, it provides algorithms that facilitate: i) the optimal planning of the quantity and the location of DERs (flexibility assets) according to the needs of various markets, and ii) the efficient scheduling of those DERs (optimal participation in and across various energy markets). All these constitute FSPs financially sustainable. It should be noted that the main difference between an ESP and an FSP is that the latter actor represents DERs in the DN which belong to its customers (i.e., end energy prosumers) and are possibly not compatible with existing energy market architectures. In contrast, an ESP owns assets in the TN which are compatible with the existing markets.

Finally, FLEXGRID develops functionalities necessary for TSOs and DSOs in the modern market architecture. In more detail, FLEXGRID’s architecture enables the interaction between DER investors (i.e. ESPs, FSPs) and system operators towards the design of more profitable investments through TN- and DN-level information exchange. Furthermore, FLEXGRID provides intelligence for network upgrades to DSO/TSOs according to the market and ESP/FSP needs. In this way, FLEXGRID guarantees that in this modern environment, malicious ESPs/FSPs will not be able to exercise market power and jeopardize market efficiency, while the DSOs/TSOs will be able to minimize their costs for future grid reinforcements.

### 2. Novel energy market architectures for integrated management of transmission and distribution network

Existing electricity markets do not consider the constraints of local distribution networks. Thus, unpredicted voltage deviations and line congestions may arise. In particular, the models used in the existing markets do not take into account power balance, power constraints and reactive power in distribution networks. As a result, re-dispatches may be necessary, which imply that: i) expensive units will have to participate in the DN dispatch and ii) higher penetration of DERs is constrained.

Novel DN models and recent advances in modern pricing algorithms allow the efficient and stable management of the DN. By exploiting such models, FLEXGRID evolves the existing energy market architectures in order to not only enable large scale and distributed RES penetration in DN, but also to increase the efficiency and the investment opportunities in modern smart grids ^2– 5^.

To this end, FLEXGRID introduces the novel concept of “Distribution Level Flexibility Market - DLFM”, which is operated by an independent entity, referred to as Flexibility Market Operator (FMO). In FLEXGRID, NODES ^6^ is the FMO that operates the proposed DLFM. In this context, FLEXGRID focuses on the development of a digital Automated Trading Platform (ATP) that facilitates FMOs to: i) operate the DLFM and interact with existing energy/balancing markets operated by MO/TSO, ii) acquire flexibility requests from DSOs, and iii) interact with ESPs and FSPs by receiving flexibility offers.

The proposed FLEXGRID energy market architectures develop, combine, and bring to interaction the following six (existing or innovative) energy markets compiled in Table 1. It should be noted that FLEXGRID follows the Nord Pool paradigm currently operating in the many European countries as EU’s regulatory baseline.

FLEXGRID developments unfold around three different flexibility market architectures. FLEXGRID puts emphasis on the trade-off between: i) social welfare maximization/ market efficiency, ii) the level of compatibility of the proposed architecture with the existing markets’ architecture (i.e., day ahead, balance), and iii) their efficiency for various energy sector stakeholders (e.g., ESPs, FSPs, market/system operators, etc.).

These three architectures are described below. The first architecture (A) is framed within existing markets (#1, #2, #5 according to Table 1), but offers the maximum possible smart grid efficiency by allowing market participants to facilitate the transmission or the distribution network at the same time, independently of their location. The second (B) acts reactively to the existing energy markets and in this way sacrifices efficiency (market participants in distribution network are not able to use transmission network and interact with wholesale market) for compatibility. The third one (C) acts proactively to the existing energy market.

**Table 1.** Summary of markets assumed within FLEXGRID.

Market #1	Market Operator (MO) operates the day-ahead energy market at the Transmission Network (TN) level
Input	MO receives bids from all market participants and basic power flow constraints at the TN level. Depending on FLEXGRID’s various energy market architectures selected from the FLEXGRID’s set of options (see below), the market clearing process of Market #1 either ignores the DN topology, or implicitly takes it into account (output of Market #3).
Output	Market clearing results (TN-aware price €/MWh per TN node and Day Ahead Dispatch (DAD)
Market #2	TSO operates the day-ahead reserve market at the TN level
Input	Receives bids from all market participants at TN level, DAD, schedules from MO, RES/demand forecasts, maintenance- related info from assets and grid, TN model/topology and constraints Depending on FLEXGRID’s various energy market architectures (see below), the market clearing process of Market #2 either ignores the DN topology, or implicitly takes it into account (output of Market #4)
Output	Reserve market clearing results at TN level (price €/MW and reserve capacity commitment per accepted market participant)
Market #3	Flexibility Market Operator (FMO) operates the day-ahead energy market at the Distribution Network (DN) level
Input	Receives bids from all market participants at DN level, and DN topology constraints. DAD schedule from MO at all TSO-DSO coupling points or respective day-ahead energy market price forecasts (depending on the selection of energy market architecture from the three FLEXGRID options below).
Output	Market clearing results (DN-aware price €/MWh and DAD per accepted market participant and DN node)
Market #4	DSO operates the day-ahead reserve market at the DN level
Input	Receives bids from all market participants at DN level, DAD schedules from FMO, local RES/demand forecasts, and maintenance-related info (if any)
Output	Reserve market clearing results at DN level (price €/MW per DN node and reserve capacity commitment per accepted market participant)
Market #5	TSO operates the balancing energy market at the TN level
Input	Receives bids from all market participants at TN level (incl. DER aggregators), updated RES/demand forecasts, updated data from SCADA/EMS. Depending on the energy market architecture selected from the FLEXGRID’s various options, the market clearing process of Market #5 either ignores the DN topology, or implicitly takes it into account through the output of Market #6.
Output	Balancing energy market clearing results at TN level (i.e. prices €/MWh per TN node, and Up/Down activation energy quantities per accepted market participant)
Market #6	DSO operates the balancing energy market at the DN level (only when DSO has a balancing responsibility for its DN operation)
Input	Receives bids from all market participants at DN level, updated local RES/demand forecasts, and updated data from DMS ^7^
Output	Balancing energy market clearing results at DN level (i.e. prices €/MWh per DN node, and Up/Down activation energy quantities per accepted market participant)

***A. A clean slate approach towards a market-based smart grid operation with optimal social welfare – Interactive DLFM (I-DLFM)***

Novel smart grid architectures which are able to maximize social welfare (through efficient markets) lead to: i) energy with lower cost for consumers, ii) more revenue streams for Energy and Flexibility Service Providers (ESPs/FSPs), and iii) lower operation/management costs for network operators (i.e. TSO and DSOs). In a smart grid with high and distributed RES and high flexibility exploitation in which the distribution network faces congestion and voltage issues, an evolved energy market architecture through an advanced interaction between MO (TSO) and FMO (DSO) is needed. In this perspective, a market architecture that evolves Markets #1, #2 and #4 and is not constrained to be compatible with their existing versions can theoretically maximize social welfare.

In the proposed Interactive DLFM (I-DLFM) model, FLEXGRID considers an iterative process that takes place between the MO and FMO until they converge to an optimal dispatch schedule at both the TN and DN levels.

**Figure 3.**
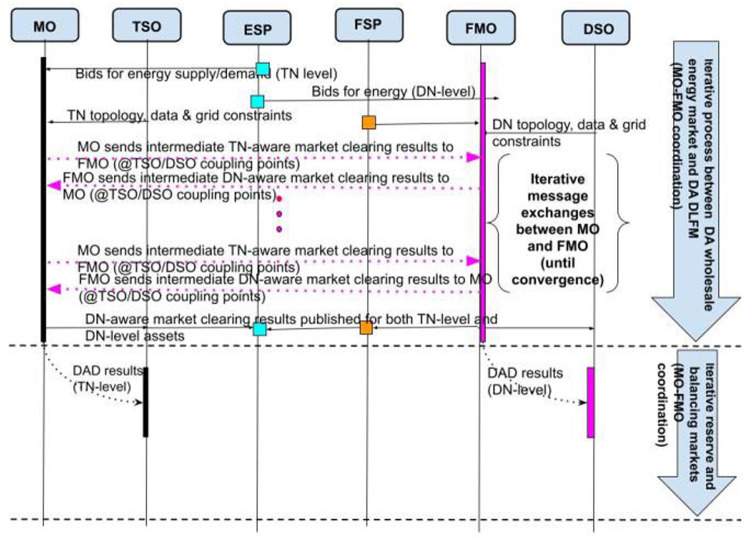
FLEXGRID’s *I-DLFM architecture*.

According to the market definitions of Table 1, FLEXGRID assumes three basic markets at both TN and DN levels: A) Day-head energy markets (interactive clearing of Markets #1 and #3), B) Day-Ahead Reserve markets (interactive clearing of Markets #2 and #4), and C) near-real-time Balancing energy markets (interactive clearing of Markets #5 and #6). For example, in the day-ahead energy market context, presented in Figure 3, the MO initially runs an instance of its market clearing problem at the TN level and sends the results to the FMO. Then, the FMO takes the MO’s results as input and runs its own market clearing problem at the DN level. The respective results (e.g., Lagrange multipliers) are sent back to the MO, who runs another round of the TN-level market clearing. Of course, the dispatch schedules that are decided in each round of the algorithm’s execution are virtual and are not actuated in reality. After several algorithmic iterations (i.e. several message exchanges between MO and FMO), the process converges (through the use of optimization theory) to an overall dispatch schedule (i.e. at both TN and DN levels) that maximizes social welfare. A similar iterative process may take place for day-ahead reserve markets and near-real-time balancing markets (cf. TSO-DSO collaboration).

**Figure 4.**
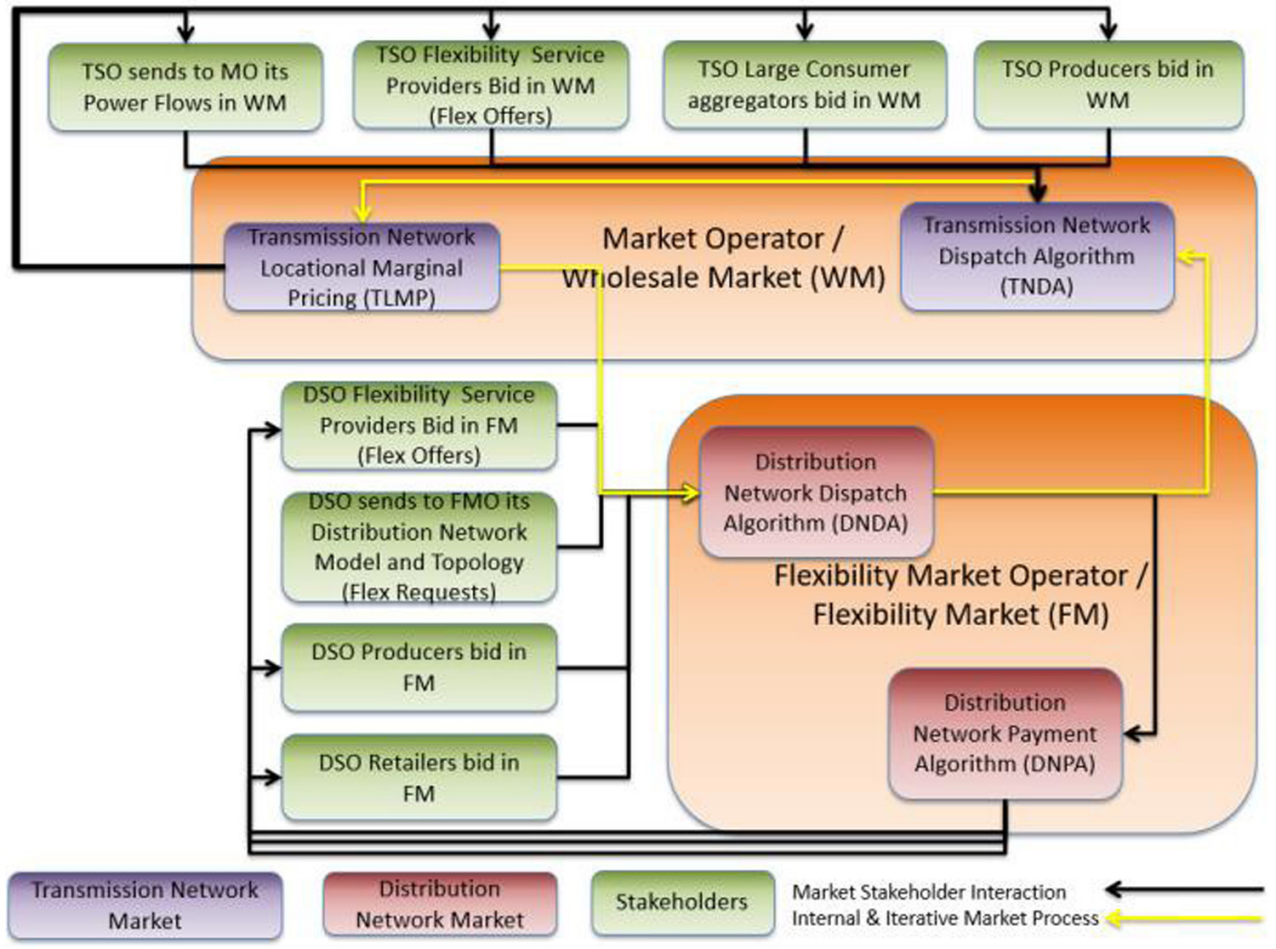
I-DLFM steps.

Figure 3 presents the aforementioned interactive market clearing process of a unified energy market, in which stakeholders in the DN (i.e., FSPs, ESPs) and the TN (ESPs) are able to trade energy. Briefly, the core of the proposed market architecture is a unified market clearing based on an iterative process (cf. purple arrows in Figure 3 and yellow arrows in Figure 4) between the MO managing the TN and the FMO managing the DN.

At each iteration of this process, and according to the bids of the TN-level market stakeholders, the MO derives a time series (according to the scheduling horizon) of prices, noted as Transmission Network Locational Marginal Prices (TLMPs) for each node in the TN. These nodes include the interface nodes through which each DN exchanges power with the TN. An FMO operating in a certain DSO area takes as input: i) TLMPs that the MO derived, and ii) the bids of the DN-level market participants. In a second step, the FMO derives a time series of power flows (Distribution Network Dispatch –DND) in each node of the DN and updates the coupling point (DN-TN connection nodes) power flow time series. The termination condition of this iterative process is an identical dispatch in TN and DN in two consecutive iterations (with respect to an accuracy threshold).

According to the final dispatch, the pricing in the TN is coherent with the existing pricing policy in today’s smart grids (TLMPs) and the pricing in the DN is conducted through a clearing algorithm that the FMO executes.

The rest of this section presents in detail the necessary steps for the operation of the proposed Interactive Market Clearing Algorithm (IMCA):

**Step 1**: The DSO sends its distribution network data to the FMO.

**Step 2**: The FSPs, connected to the DSO, send their flexibility asset (e.g. ESS, DSM) bids (i.e. FlexOffers) to the FMO. Each FlexOffer includes the cost/utility function of the FSP and its operating constraints.

**Step 3**: The Producers (ESPs) connected to distribution network (e.g. RES, prosumers) send their bids to the FMO.

**Step 4**: The Consumers (ESPs) connected to the distribution network (i.e., demand aggregators) send their bids to the FMO.

**Step 5**: The MO generates a forecast of the TLMPs for the first iteration of the IMCA

**Step 6**: In each iteration k of MCA

**Step 6a**: Taking the TLMP _k_ (TLMPs in k ^th^ iteration of IMCA) at the coupling point, the FMO generates a Distribution Network Dispatch denoted by DND _k_ (k ^th^ iteration of IMCA) for each specific DSO area through the execution of the DND Algorithm (DNDA).

**Step 6b**: The DLFM stakeholders are compensated for their operation according to a Distribution Network Payment Algorithm (DNPA). A detailed discussion of the DNDA and DNPA requirements and a description of the related FLEXGRID developments is included in the Section IV.

**Step 6c:** The TN stakeholders (i.e. generators, demand aggregators, etc) decide their dispatch based on the corresponding TLMP _k_. The TSO calculates its power flows based on the zonal TLMP _k_, which along with the TN-level stakeholders and the FMOs’ decisions formulate the TN Dispatch (TND).

**Step 6d**: If the DND and the TND remain the same between two consecutive iterations of IMCA, then IMCA terminates. Otherwise, TLMP _k_ are calculated by a TLMP Update Algorithm (TLMP-UA) based on the previously computed dispatches and TLMP _k_). FLEXGRID develops various TLMP-UAs mainly based on duality theory ^7^ and decomposition techniques ^8^ to guarantee their convergence.

**Step 7**: The last calculation of TLMPs and the last calculation of DNPA determine the payments of participants in the TN and DN, respectively. The last TND and DND solutions/schedules determine the dispatch in the two networks.

As this market is incompatible with the existing energy market architectures, FLEXGRID implemented this market in simulations in order to realize its advantages and quantify the disadvantages (through comparisons) over the two other markets that are proposed below (reactive and proactive DLFM).

***B. A wholesale market-compatible and distribution network-aware market – Reactive DLFM (R-DLFM)***

In contrast to the I-DLFM, the objective of the R-DLFM architecture is to be compatible and capable to interact with the existing TN-level markets (cf. #1, #2 and #5 in Table 1). In case that the R-DFLM operates right after day ahead market, it is capable to deal with: i) congestion issues at DN level that DAD cannot capture, and ii) forecast inaccuracies in energy production and energy consumption of DN- and TN-level assets.

The drawback of this approach is the possibility of infeasibility, or the need for mandatory/forced curtailments, or financially unsustainable distribution of DAD due to costly flexibility assets. All these may lead to discontented producers/consumers. Furthermore, in cases in which DAD is modified, the spot market price (cf. market #5) at the transmission level have to be paid. Finally, the absence of joint optimization between transmission and distribution levels leads to lower market efficiency, which in turn deteriorates the economic viability of the participating stakeholders.

**Figure 5.**
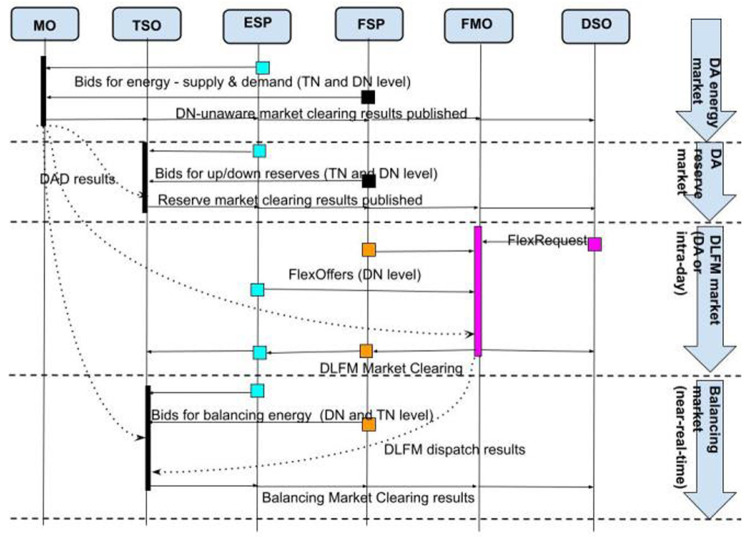
FLEXGRID’s R *-DLFM architecture*.

The sequence and timing of the markets in the R-DLFM energy market architecture is described in Figure 5. Initially the operation of the Day Ahead (#1) and the Reserve Market (#2) take place. Then FMO takes the output of these two markets and clears the R-DLFM (#3) according to: i) DSO’s network topology and constraints (which generate FlexRequest) and ii) FSP and ESP bids (FlexOffers). A reserve market at DN level could optionally take place in this phase through the same procedure (#4). In the next phase the outputs of DAD and R-DLFM are given as input to the Balancing Market (#5). In the optional case that DSO wants to handle voltage limits due to RES in DN a balancing energy/capacity market at the DN level (#6) may also take place right after clearing the TN-level balancing energy market.

**Figure 6.**
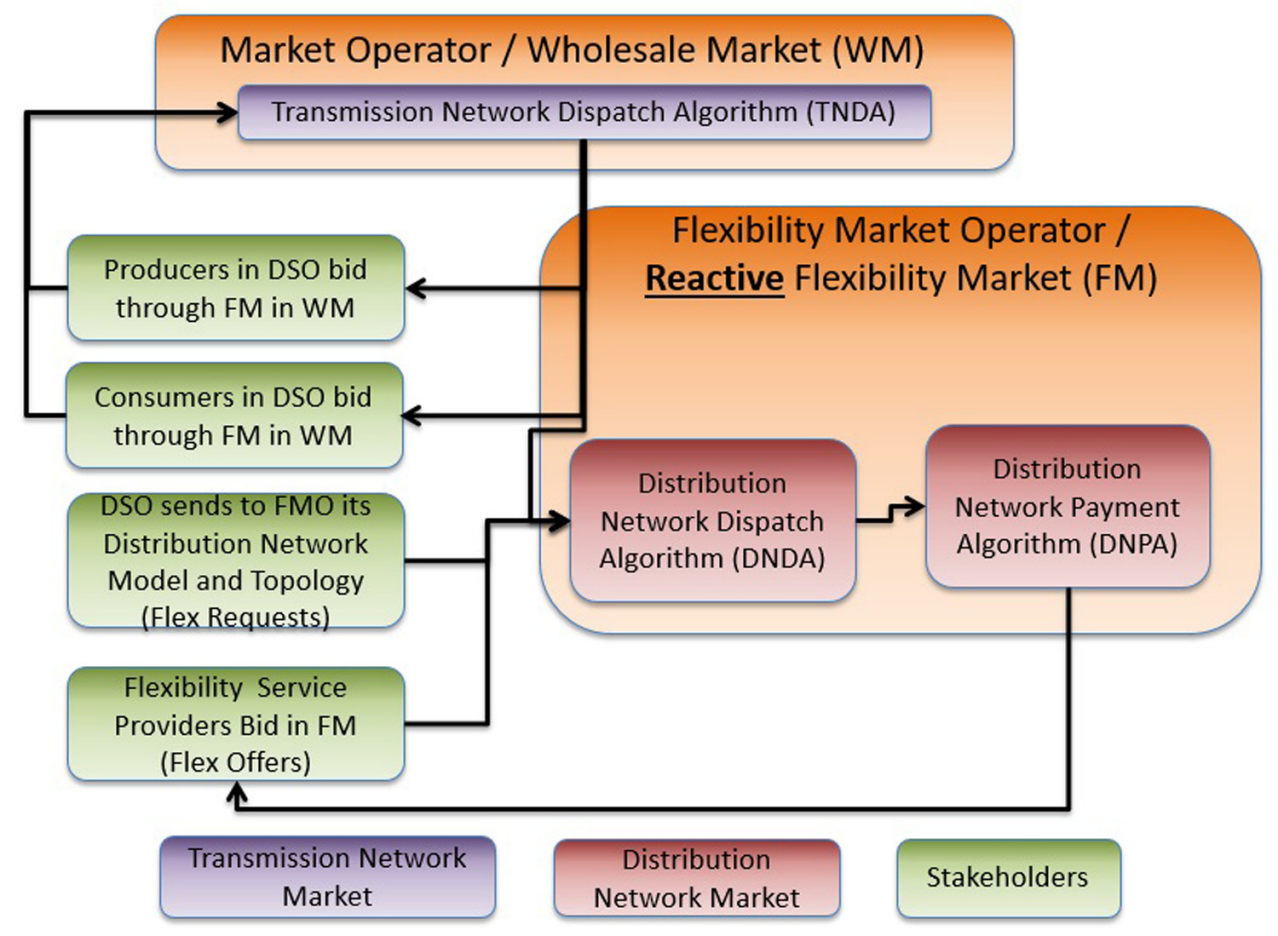
R- *DLFM* steps.

Figure 6 illustrates the steps of the R-DLFM market clearing process:

**Step 1**: The FMO takes as input the DAD that is composed from the power flows in the coupling point with the TSO and the dispatch that concerns producers and consumers in its distribution network.

**Step 2**: The DSO sends its DN data to the FMO.

**Step 3**: The FSPs and ESPs connected to the DSO send their flexibility bids to FMO.

**Step 4**: The FMO generates DND (execution of a DNDA).

**Step 5**: Flexibility assets are compensated for their operation according to a DNPA that the FMO executes.

***C. Feasibility check and optimization of wholesale market bidding through a proactive distribution network-aware market - Proactive DLFM (P-DLFM)***

In order to mitigate the drawback of the aforementioned architecture (namely, the difficulty to manage an infeasible (DN level) or expensive (TN level) market clearing), FLEXGRID proposes a proactive clearing of bids in the DN by the FMO before the MO clearing. In this way, it ensures an a-priori feasible dispatch of the DN-level DERs.

In order to allow the FMO to operate proactively, an accurate estimation of the TN-level market clearing prices (Markets #1, #2 and #5) at the TSO-DSO coupling point is required. An underestimation in TLMPs may result in a lower demand than calculated by TN level markets. An overestimation in TLMPs may lead to an over-supply of generation dispatch.

**Figure 7.**
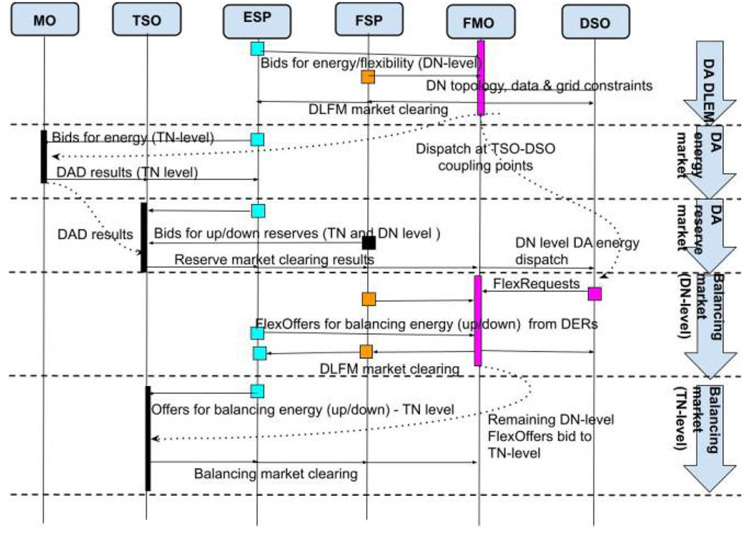
FLEXGRID’s P- *DLFM architecture*.

As shown in Figure 7, the sequence of markets starts with Market #3 operated by the FMO. In this phase, the P-DLFM is a day-ahead energy market at the DN level (ESPs and FSPs in DN level bid in this market). Right afterwards, in the next phase, the Day-Ahead Market (#1) and the Day-Ahead Reserve Market (#2) at TN level close. The output of Day-Ahead P-DLFM acts as input to the Day-Ahead market at TN level. Finally, for near-real-time balancing markets, the DSO runs a proactive balancing market (Market #6) right before the established balancing capacity market operated by the TSO (Market #5). Thus, the local congestion and voltage problems at the DN level can be directly solved by the DSO locally, at the DN level, while the “remaining bids” of “Balancing P-DLFM” (Market #6) can be used as input to the Balancing Market (Market #5). The Balancing P-DLFM acts as a balancing market at DN level and takes as input ESP and FSP bids at that level. It additionally propagates its clearing prices (output) into the TN level balancing market which may facilitate balancing at DN level in case of inadequate balancing resources.

**Figure 8.**
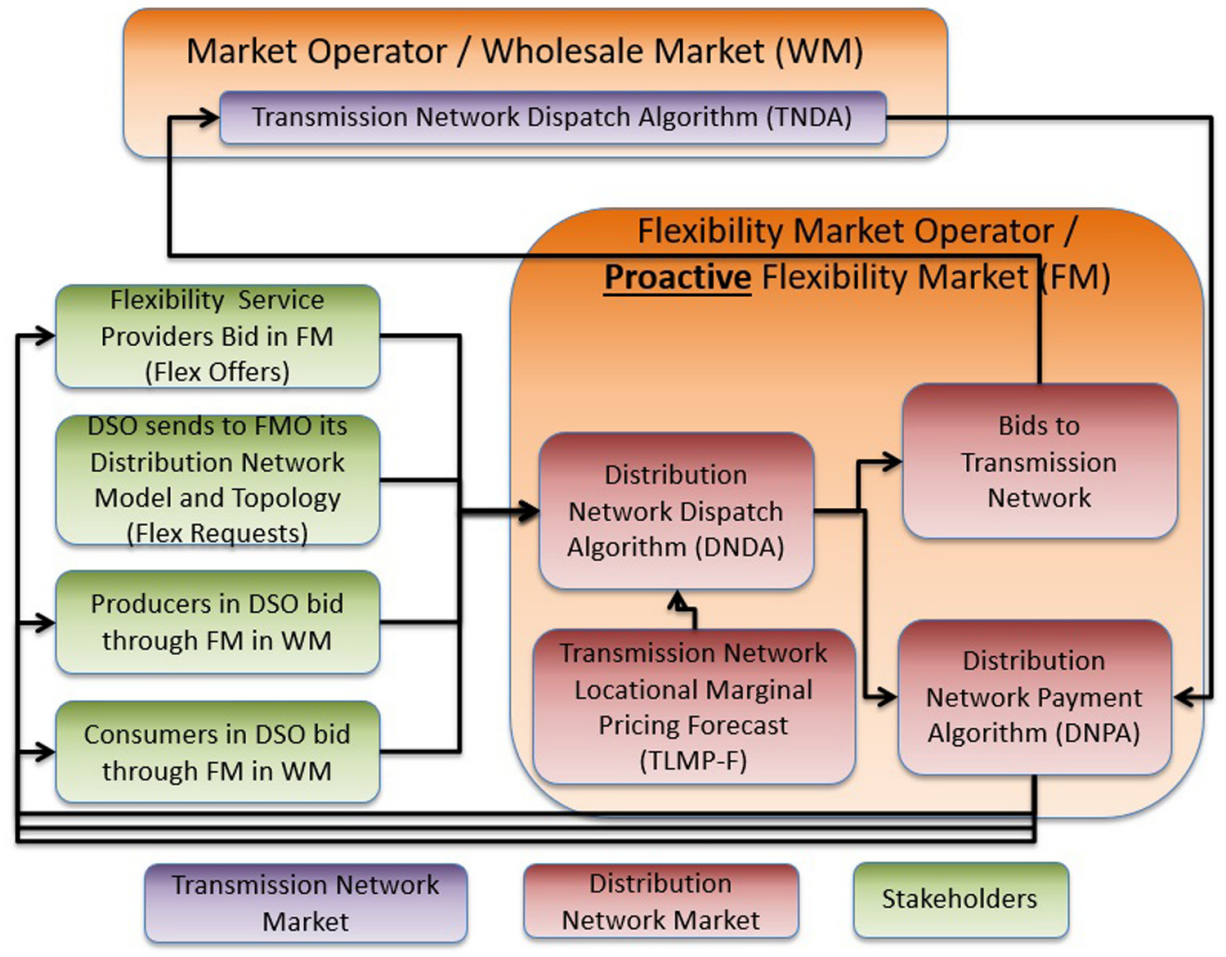
P-DLFM steps.

Figure 8 shows the steps of the P-DLFM market clearing process and its interaction with TN level markets:

**Step 1**: The DSO sends to the FMO information that suffices to model its distribution network.

**Step 2**: The FSPs connected to the DSO send their flexibility bids to the FMO.

**Step 3**: The Producers (ESPs) connected to the DSO send their production bids to the FMO.

**Step 4**: The Retailers (ESPs) connected to the DSO send their consumption bids to the FMO.

**Step 5**: The FMO (or any other party) generates a forecast for the TLMPs for the coupling point at which the DSO is connected.

**Step 6**: The FMO generates the DND through the execution of an algorithm noted as DNDA (may reduce the quantity in the initial bids, but it does not reduce the bidding prices).

**Step 7**: The TLD uses DNDA results as input bids.

**Step 8**: After the TLD, the DNPA uses as input: i) bidding prices of flexibility providers, producers and consumers, ii) TLD, and iii) DLD in order to derive the compensations for all the stakeholders that bid in Steps 2-4.

### 3. Operation

This subsection explains the basic steps that an interested user, S/W developer or researcher should follow in order to be able to download, install and configure a FLEXGRID service in its own system. Due to the modular-by-design FLEXGRID ATP architecture, each FLEXGRID ATP service can be offered as a stand-alone service or as a part of a bunch of services according to the end customer’s business preferences. FLEXGRID ATP deployment is based on open source S/W tools and thus FLEXGRID services are publicly available in the project’s GitHub area. The S/W installation and configuration steps can be summarized as follows:

**Step 1:** Design your API using swagger editor

**Step 2:** Connect to FLEXGRID Central Database

**Step 3:** Deploy, test and run your server locally

**Step 4:** Deploy the FLEXGRID application on your server

**Step 5:** Implement the UCS DES-B3 algorithm (or whichever other FLEXGRID service/algorithm)

**Step 6:** Use external data or sample data to further test and validate the algorithmic operation

It should be noted that in this position paper, we describe the configuration steps for FLEXGRID DES-B3 (see more details about this Digital Energy Service below), while the same set of steps should be followed for all other FLEXGRID services, too. For more technical details, the interested reader should follow the relevant instructions in the user manual (cf. readme online file in FLEXGRID GitHub area).

The requirements to run FLEXGRID are a Linux server running Python 3.8.10 or above, and with the following Python packages:

- connexion==2.6.0
- glpk==0.4.6
- ipython==7.24.1
- ipython-genutils==0.2.0
- numpy==1.20.3
- Pyomo==6.0.1
- python-dateutil==2.8.1
- python-dotenv==0.17.1
- requests-mock==1.9.3
- responses==0.13.3

Additionally, FLEXGRID requires a web server that supports uWSGI such as NGINX.

## III. Use cases

In this section, we present Digital Energy Services (DES) which can be seen as the use cases that complement and constitute efficient the proposed flexibility market architectures as analysed above. More specifically, they facilitate the operation of FSPs, ESPs, and System Operators (DSOs/TSOs) and we provide references to our published work for more detailed analysis.

### A. DESs for FSPs and efficient end user flexibility aggregation

In order to facilitate the efficient interaction of FSPs (which FLEXGRID examines as a specific ESP category related with DN and end user flexibility management) with modern smart grids, FLEXGRID develops a set of DESs to automate this operation. These DESs can be used for: i) the efficient aggregation of flexibility assets that end users dispose at a specific DN location and ii) the optimal exploitation of them in the complex and emerging energy market architecture. The DESs to FSPs that FLEXGRID develops unfold around three major use cases, which are analyzed in the rest of this section.

***DES-A1: Automated, efficient and dynamic aggregation of flexibility assets through advanced retail pricing models and auction-based mechanisms.***

The aggregation of small-scale distributed flexibility assets (end user electric appliances with controllable loads, electric vehicles, batteries, etc.) requires the development of an advanced retail market through which FSPs trade dynamically with end energy prosumers (or else end users). DES-A1 takes as input: i) flexibility needs (flexibiltiy market prices) , ii) flexibility asset availability and iii) preferences of the end users and through dynamic pricing schemes and auction-based processes. It produces as output the flexibility asset aggregation and the compensation to the end users. The dynamic pricing schemes and auction-based processes that FLEXGRID develops are: i) real time and scalable, in the sense that they have the ability to find solution within acceptable (specified requirement) time limits (FLEXGRID considers both day-ahead and near-real-time cases), ii) efficient and able to provide effective flexibility exploitation ^9, 10^, iii) strategy-proof, in the sense that they do not allow the manipulation of flexibility aggregation by a set of malicious end users ^11, 12^, iv) competitive, in order to allow FSPs to provide competitive prices to their end users ^13^, v) fair in terms of compensating end users according to the market value of their assets ^14^, and vi) privacy protecting of the end user’s personal data ^11^. Moreover, the uncertainty in the constraints and preferences that the end user introduces is a critical research thread towards development of advanced pricing schemes.

More specifically, FLEXGRID develops an Automated Flexibility Aggregation Toolkit (AFAT). AFAT integrates several retail market pricing schemes and auction algorithms (that deliver various trade-offs of the requirements above). In exercising this business approach, FSPs can utilize advanced forecasting services to predict market prices and net load profiles of available end users’ assets. Thus, FSPs optimally use the available flexibility (by dynamically adapting retail pricing) and in this way maximize the total profits for all participants in the FSP’s portfolio.

***DES-A2: Develops advanced RES and market forecasting ESs that predict the prices in the markets, thus facilitating FSPs to efficiently compose and exploit their portfolio (i.e. flexibility assets).***

This DES includes forecasting of RES’ aggregated generation and forecasting of market prices. DES-A2 takes as input: i) history of the market prices, ii) history of RES production/demand and iii) weather info and through forecasting algorithms. It produces as output estimations for RES production and market prices. Without lack of generality, FLEXGRID assumes aggregator’s participation in both TN- and DN-level markets. Thus, forecasts will be applicable to all these markets (which are existing or FLEXGRID’s developments) in order to allow FSPs to efficiently derive their assets’ scheduling.

***DES-A3: Efficient exploitation of FSP’s portfolio by optimally interacting (maximize FSP’s revenues) with several energy markets according to the FLEXGRID innovative energy market architecture.***

FSPs aggregate flexibility assets from end users and trade their flexibility asset portfolio in various energy markets. DES-A3 takes as input: i) flexibility asset portfolio and ii) market forecasts and bidding history. It produces as output the aggregated flexibility asset dispatch and the compensation to the FSPs. The innovative portfolio scheduling that FLEXGRID develops ^15, 16^ as a DES to the FSPs takes into account: i) detailed models of all the DERs that they may dispose, ii) their simultaneous participation in all energy markets (i.e. according to the three architectures that are analyzed above), iii) the existing competitors of flexibility aggregators and their dynamic behavior/strategy. The improvements through this DES are: i) the financial sustainability of the flexibility asset owners (i.e. end users) through more market opportunities and more efficient use of their assets, ii) the decrease of the smart grid management costs (network operators, which interact with FSPs, are able to exploit enhanced liquidity of flexibility assets in order to manage their networks with lower cost).

### B. Advanced DER exploitation for Energy Service Providers (ESPs)

In order to facilitate third parties to efficiently interact with modern smart grid markets and provide competitive services through them, FLEXGRID equips ESPs with management services able to: i) optimally operate their available DER (e.g., RES, energy storage systems-ESS, demand side management-DSM), and ii) optimally plan their investments on them.

The various Business Models (BMs) that FLEXGRID serves are categorized according to the available data that the ESPs dispose. The basic BM assumes only knowledge of the market price forecast. More advanced BMs assume operation and investments in DERs are also based on the knowledge of: i) the topology and dynamic traffic in TN and DNs, and ii) the strategies of possible ESP competitors.

Management services to ESPs that FLEXGRID develops unfold around six major DESs, which are analyzed in the rest of this section.

***DES-B1: Minimization of ESP’s Operational Expenditures (OPEX) by co-optimizing: i) the consumption of its end users (DSM), ii) the production of its RES and iii) the operation of its ESS.***

In this innovative market landscape, ESPs seek to optimize their assets’ portfolio in order to maximize their profit and/or the welfare of their end users, by participating in a number of energy markets. DES-B1 takes as input: i) available DERs, ii) DSO topology and iii) transmission level market forecasts. It produces as output the dispatch of DERs, the way that they will participate in various markets and the compensation of each DER operator. Generally, the DERs operated by the ESPs are: flexible loads, inflexible loads, ESSs and production from RES. DER management DESs on the ESP side aim at a sophisticated interaction with network operators (DSO/TSO) through the competitive modern market architectures that FLEXGRID proposes. In this highly competitive landscape, ESPs determine the strategy that maximizes their profits and/or the welfare of their end users by optimally scheduling: i) their market actions (buying/selling), and ii) the operation of their DERs (e.g. ESS/EV charging/discharging, shifting/curtailing of flexible loads, RES usage, etc.).

***DES-B2: ESP’s optimal investment strategies in DERs***

The formation of an ESP’s portfolio, which considers the quantity and the network location (i.e., sizing and siting) of its DERs related with capital investments are long-term decisions ^5^. DES-B2 takes as input: i) available DERs, ii) DSO topology and iii) long term transmission level market and demand forecasts. It produces as output the quantity and the network location (i.e., sizing and siting) of DERs that will be installed. Consequently, the modelling of this portfolio requires multi-stage planning and high robustness against potential inaccuracies in predictions regarding: i) the future market trends, ii) demand curves, iii) weather conditions, and iv) other variables that could influence ESP’s market position.

FLEXGRID develops DESs that offer advanced investment strategies to ESPs that are adaptable to the desirable robustness level and the information disposed by the ESP, such as: i) market price forecasts, ii) the topology and dynamic traffic flows in the transmission and the distribution networks, and iii) the strategies of the ESP’s competitors.

***DES-B3: Co-optimization of DER operation that an ESP disposes towards maximum aggregated profits through its parallel participation in multiple DN- and TN-level markets***

Congestion management and frequency/voltage control issues caused by high RES penetration increase the volatility of energy prices in modern energy markets (TN-level) as well as in emerging local flexibility markets (DN-level). This temporal and spatial volatility reveals the markets’ characteristics and in the same time offers business opportunities and revenues for ESPs that invest on DERs ^3^.

In order to be sustainable and competitive, ESPs should maximize their aggregated profits by dynamically co-optimizing their bidding strategy across several energy markets. FLEXGRID not only models the existing markets (Markets #1, #2, #5), but also models emerging DLFMs (Markets #3, #4, #6). In this way, the ESPs are able to select their own BM, which determines the markets in which they are allowed to participate, based also on the existing regulatory framework. DES-B3 takes as input: i) available DERs, ii) DN- and TN-level market forecasts. It produces as output the dispatch of DERs and the compensation of each DER operator.

***DES-B4: ESP that manages the distribution network and the DERs attached to it.***

This DES concerns ESPs that may be microgrid operators, energy cooperatives, energy islands with weak or no connection to the main grid, etc. In this case, there is no interaction between the ESP and the local market (e.g., Markets #3, #4, #6) because the ESP not only manages a DER portfolio, but it simultaneously manages the distribution network, as analyzed in 4, 17. Hence, the ESP’s objective is the optimal scheduling of its DERs and its optimal interaction with the existing energy markets (Markets #1, #2, #5) towards maximization of its profits or maximization of the welfare of its end users (i.e., DER owners). DES-B4 takes as input: i) available DERs, ii) DSO topology and iii) demand. It produces as output the optimal dispatch of DERs.

***DES-B5: ESPs that are primarily RES Producers (RESPs).***

This use case is similar to DES-B1. It concerns the operation of hybrid RES/ESS systems and their participation in the energy market architecture. More specifically, a RESP actor is essentially an ESP owning DERs that are mainly RES (large photovoltaic and/or wind parks). DES-B5 aims at optimizing combined RES/ESS systems in order to avoid the imbalance penalties that are included in the optimization model. The proposed DES allows RESPs to: i) monitor, analyze and predict RES generation and market prices towards more efficient use of their assets, ii) plan and operate their assets optimally towards more competitive BMs and increased revenues in the modern market architectures proposed by FLEXGRID. DES-B5 takes as input: i) available RES, ii) DSO topology and iii) transmission level market forecasts. It produces as output the dispatch of RES and their compensation.

***DES-B6: ESPs that are mainly ESS owners or that lease ESS for several purposes to several stakeholders simultaneously.***

The participation of numerous DERs, providing non-negligible total volume, are one of the main prerequisites for the flexibility market to function in its full efficiency. There are complex BMs for managing and generating income from the use of DERs. DES-B6 facilitates this type of ESPs to operate financially sustainable BMs, with respect to the aforementioned energy markets, by leasing DERs. More analytically, this DES focuses on an ESP, which acts as an ESS owner and leases its ESS to various stakeholders that are market participants in several markets. DES-B6 takes as input: i) available DERs, ii) DN- and TN-level market forecasts and TL services that request flexibility supply. It produces as output the optimal participation of ESS (bidding) in each market and/or flexibility supply request.

### C. Advanced System/Network DES provision for Network Operators (DSOs/TSOs)

TSOs and DSOs are service-oriented companies responsible for the transmission and distribution of electricity, respectively. In an era of high and distributed RES penetration, the management of transmission and distribution in a robust and efficient way constitutes the raison d’etre of modern smart grids.

In order to achieve these, TSOs have to interact very dynamically with DSOs and ensure the stability of their networks. In addition, both have to interact with profit-based DER investors in a way that they facilitate the efficiency of ESPs’ investments or else guarantee the stable grid operation with the lowest possible cost for flexibility. In parallel, selective upgrades in the topology of their networks should be performed to mitigate market power strategies of malicious ESPs and ensure the optimization of social welfare (low cost of energy services and improved profitability of non-malicious ESPs).

***DES-C1: Flexibility asset investments in which a System Operator shares information with an ESP (DER investor) towards more attractive trade-off between transmission/distribution costs and investment’s financial sustainability.***

In order to secure stability/reliability and low cost of the future smart grid, this DES ^18^ develops long-term DER investment planning while considering factors such as: i) underlying network topology, and ii) topology-aware production/demand curves. Traditionally, the most common choice for securing sufficient network capacity during the peak hours is expansion of the network using physical assets, i.e., new lines, transformers, circuit breakers, etc. This method has two negative consequences: i) high capital expenditure (CAPEX), ii) transmission/ distribution system is most of the time heavily under-capacitated.

A different perspective is the smart placement and utilization of DERs, which can alleviate/postpone major network expansion investments and result in robust transmission and distribution systems. FLEXGRID further evolves this strategy by offering DES to system operators through which they can accurately inform DER investors to conduct investments adapted to their economic viability problems. DES-C1 takes as input: i) available DERs and bidding history, ii) DSO/TSO topology and iii) long term TL and DL market and demand forecasts. It produces as output the quantity and the network location (i.e., sizing and siting) of DERs that will be installed.

***DES-C2: TSO-DSO collaboration for coordinated and dynamic management of aggregated flexibility assets.***

Customers and distributed third-party energy resources that have the ability to adjust their consumption, generation, or storage units in short time could be aggregated and their flexibility could be offered directly as ancillary service to the TSO or to be used for DSO purposes. Thus, ancillary services like active/reactive power control could take place more efficiently through an efficient exchange of flexibility between TSO and DSO.

The main innovation here is to supply reliable and efficient flexibility to TSO or DSO from geographically distributed third-party energy resources. According to the proposed collaboration model, the flexibility assets that TSOs and DSOs directly control in order to handle very dynamic and short-term stability can be used to help each other. In this way, these DES become much more dynamic and efficient. DES-C2 takes as input: i) available DERs, ii) DSO/TSO topology and iii) demand forecasts. It produces as output the dispatch of DERs in DN and TN.

***DES-C3: Market power aware upgrade of DN and/or TN.***

The development of dynamic DLFM (e.g. Markets #3, #4, #6) facilitates high RES penetration by increasing the liquidity of DERs, thus reducing the cost of energy. At the same time, in contrast with traditional grid architectures in which flexibility is managed in a direct control fashion from the system operator, smart grids “acquire” a vulnerability relevant with the way that strategic and malicious ESPs may affect/manipulate the market prices.

In order to mitigate its effects, FLEXGRID offers ESs through which TSOs and DSOs are able to upgrade their network by taking into account information relevant to the market power aware network topology weaknesses. In accordance with this DES, topology aware flexibility investments further mitigate market power and in this way system operators and end users are able to experience flexibility markets’ benefits. DES-C3 takes as input: i) available DERs and bidding history, ii) DSO/TSO topology and iii) long term TL and DL market and demand forecasts. It produces as output the quantity and the network location of DN and TN upgrades.

## IV. Conclusions

In this work we presented a novel smart grid architecture and novel flexibility markets that constitute its orchestration functionality. In addition, we presented novel Digital Energy Services critical for the efficiency and the smooth operation of the proposed architecture.

FLEXGRID’s plan and future work is to compare the aforementioned energy market architectures and evaluate its proposed algorithmic toolkit using real data by focusing on: i) market clearing and pricing algorithms, and ii) DER operation and planning algorithms. Another major goal of FLEXGRID is to provide a comprehensive regulatory and policy framework roadmap to the European Commission by offering recommendations regarding the introduction of DLFMs in the existing energy markets and grid operations. In this way, FLEXGRID will not only offer a range of proposed architectures to future smart grids, and a description of the best version of each of them, but will also quantify their strengths and weaknesses. This will allow EU policy makers/regulators to harvest the potential gains and avoid the risks in the design of the clean energy transition.

## Software availability

Software available from: https://flexgrid-project.eu/github.html

Source code available from: https://github.com/FlexGrid/FST-service-3-stacked-revenues-maximization

Archived source code at time of publication: https://doi.org/10.5281/zenodo.5567010 ^19^

License: GNU General Public License version 3

## Data availability

No data are associated with this article
