# Supplementary material for: FLEXGRID – A novel smart grid architecture that facilitates high-RES penetration through innovative flexibility markets towards efficient stakeholder interaction
Source: Open Res Eur. 2023 Oct 2;1:128. Originally published 2021 Oct 22. [Version 2] doi: 10.12688/openreseurope.14109.2 (PMC10663655; doi:10.12688/openreseurope.14109.2)
Supplement: Peer review history

# Reviewer report: Reviewer response for version 2

**DOI:** 10.21956/openreseurope.17941.r35986

**Published:** 2023-11-21

**Authors:** Sonam Norbu

**Affiliations:** 1 University of Glasgow, Glasgow, Scotland, UK

This paper provides an innovative market framework for smart grids, emphasizing stakeholder interaction and flexibility markets. The proposed FLEXGRID market architecture shows a potential to establish a promising framework for smart local energy systems.

 While the conceptual strength is evident, a minor comment from a reviewer, although it is specifically mentioned in the future works, reviewer strongly recommend to include relevant case-studies and results to compliment the proposed FLEXGRID framework.

Is the rationale for developing the new software tool clearly explained?

Yes

Is the description of the software tool technically sound?

Yes

Are sufficient details of the code, methods and analysis (if applicable) provided to allow replication of the software development and its use by others?

Partly

Is sufficient information provided to allow interpretation of the expected output datasets and any results generated using the tool?

Yes

Reviewer Expertise:

Energy Communities, Smart Local Energy System, Smart Grid, and Local Place-based Decarbonisation

I confirm that I have read this submission and believe that I have an appropriate level of expertise to confirm that it is of an acceptable scientific standard.

# Reviewer report: Reviewer response for version 2

**DOI:** 10.21956/openreseurope.17941.r35426

**Published:** 2023-10-16

**Authors:** José Pablo Chaves Ávila

**Affiliations:** 1 " Institute for Research in Technology (IIT), ICAI School of Engineering", Comillas Pontifical University, Madrid, Spain

Thank you for addressing my previous comments, most of them were solved. I still have some remarks:

1. Figure 1 is still not clear to me, please clarify what is the meaning of the arrows.
2. You claim that "for near-real-time balancing markets, the DSO runs a proactive balancing market (Market #6)". what is the purpose of this market? This is critical because the TSOs are in charge of balancing markets. If you refer to the DSO market close to real-time to balance their system or to dispatch resources to solve network constraints that have to be clarified.

Is the rationale for developing the new software tool clearly explained?

Yes

Is the description of the software tool technically sound?

Yes

Are sufficient details of the code, methods and analysis (if applicable) provided to allow replication of the software development and its use by others?

Yes

Is sufficient information provided to allow interpretation of the expected output datasets and any results generated using the tool?

Yes

Reviewer Expertise:

Electricity market designs. Power system Economics. Demand-side flexibility.

I confirm that I have read this submission and believe that I have an appropriate level of expertise to confirm that it is of an acceptable scientific standard, however I have significant reservations, as outlined above.

# Reviewer report: Reviewer response for version 1

**DOI:** 10.21956/openreseurope.15210.r28038

**Published:** 2021-12-03

**Authors:** Antonis Papanikolaou, Georgios Pitsiladis

**Affiliations:** 1 Hypertech Energy Labs, Hypertech SA, Chalandri, Greece

This paper presents an innovative market framework where digital energy services facilitate the efficient stakeholder interaction and the cooperation of flexibility markets in smart grids. In the context of FLEXGRID, three different flexibility market architectures have been designed and developed. The purpose of these market architectures is to enable the interaction of different energy stakeholders to enhance smart grid efficiency and profits for the market participants. The authors evaluate each one of the architectures based on the potential efficiency and the compatibility with the existing market’s schemes. The market clearing process of each architecture has been developed as a FLEXGRID service and is accessible by any interested user. In addition, various uses cases and business models complementing the proposed solutions are provided.

 Overall, the proposed market framework is very interesting and its contribution for the future integration of energy markets may be significant. The paper is overall well written and easy to follow. Some comments for further consideration are provided in the following paragraphs.

 Figure 1 is not very clear. Perhaps it is necessary to elaborate a little bit more the interactions between the market actors and the functionalities as well as how FLEXGRID components facilitate each stakeholder especially DSOs and TSOs.

 Regarding the proposed market architectures, their applicability and compliance to current or expected – based on the Electricity Market Directive - EU regulatory frameworks is not evident from the article. A very brief analysis would assist the reader to understand the true potential of the proposed architectures.

 Is it possible to apply these markets frameworks where several local DSOs are connected to different TN nodes of the TSOs’ network? If possible, how many Flexibility Market Operators will exist and how will interact with the Market Operator. Some further analysis in this direction will showcase the applicability of the proposed architectures in electricity systems with different network and market configurations.

Is the rationale for developing the new software tool clearly explained?

Yes

Is the description of the software tool technically sound?

Yes

Are sufficient details of the code, methods and analysis (if applicable) provided to allow replication of the software development and its use by others?

Yes

Is sufficient information provided to allow interpretation of the expected output datasets and any results generated using the tool?

Yes

Reviewer Expertise:

Energy transition, energy demand management, energy markets

We confirm that we have read this submission and believe that we have an appropriate level of expertise to confirm that it is of an acceptable scientific standard.

# Reviewer report: Reviewer response for version 1

**DOI:** 10.21956/openreseurope.15210.r27899

**Published:** 2021-11-22

**Authors:** José Pablo Chaves Ávila

**Affiliations:** 1 " Institute for Research in Technology (IIT), ICAI School of Engineering", Comillas Pontifical University, Madrid, Spain

This paper presents an advanced market framework for flexibility services. The framework presents an open source model which considers the different operation and investment decisions from different agents. The contribution of the framework is significant and very relevant for different projects which address this topic.

 There are some aspects of the presented framework which are not clear from a regulatory point of view. Some discussion and regulatory recommendations on the different roles considered and the function the agents perform provide good insights.

 In the introduction, when referring to “inefficient investment planning and management of DER assets”. It is not clear who is the planner of DERs. Planning is generally related to network and regulated companies. DERs’ owners take investment decisions according to diverse economic signals including market prices, tariffs, self-generation regulations, etc. I recommend rephrasing this to avoid confusion. What are the roles of Energy Service Providers (ESP)?. Can ESP perform aggregator aggregator? It seems that they own DERs, but does it consider also resources inside customer premises?

 Figure 1 is difficult to read: what are the arrows indicating? Money flow?

 The MO is responsible for the efficient and network-aware market clearing at TN-level. Is it the TSO? It would be good to specify the roles of each agent.

 You state that in market #4 “DSO operates the day-ahead reserve market at the DN level”. Are these operating reserves for frequency control or availability reserves for congestion management?. I believe the latter.  

 In market #6, “DSO operates the balancing energy market” the balancing energy market is the responsibility of the TSO, not of the DSO. Can you clarify or comment on the regulatory implications for this market? Is this proposal possible in the European market model?

  Figure 7 needs further explanation to understand the links, colours, etc.

 For ESPs, you consider the network location. How is this made in practical terms? Are you proposing to disclose network data? If so, in which format and what data? I recommend mentioning the practical implementation challenges.

Is the rationale for developing the new software tool clearly explained?

Yes

Is the description of the software tool technically sound?

Yes

Are sufficient details of the code, methods and analysis (if applicable) provided to allow replication of the software development and its use by others?

Yes

Is sufficient information provided to allow interpretation of the expected output datasets and any results generated using the tool?

Yes

Reviewer Expertise:

Electricity market designs. Power system Economics. Demand-side flexibility.

I confirm that I have read this submission and believe that I have an appropriate level of expertise to confirm that it is of an acceptable scientific standard, however I have significant reservations, as outlined above.
